# Maternal Prenatal Folic Acid Supplementation Programs Offspring Lipid Metabolism by Aberrant DNA Methylation in Hepatic *ATGL* and Adipose *LPL* in Rats

**DOI:** 10.3390/nu9090935

**Published:** 2017-08-26

**Authors:** Xue Yang, Yifan Huang, Changhao Sun, Jie Li

**Affiliations:** National Key Discipline, Department of Nutrition and Food Hygiene, School of Public Health, Harbin Medical University, Harbin 150086, China; xue_yanghmu@yahoo.com (X.Y.); yifan_huanghmu@yahoo.com (Y.H.)

**Keywords:** folic acid supplementation, lipid metabolism, DNA methylation

## Abstract

The effects of maternal prenatal folic acid supplementation (FAS) on offspring lipid metabolism in adulthood remains unclear, although prenatal FAS is compulsively suggested in many countries. Female Sprague-Dawley rats were fed with control (CON) or FAS diets before and during pregnancy. Male offspring of CON and FAS dams were further divided into two groups at seven weeks for CON and high-fat (HF) diet interventions for eight weeks in adulthood (*n* = 10). The interactive effects of maternal prenatal FAS and offspring HF in adulthood on lipid metabolism and DNA methylation of genes involved in lipids metabolism were assessed. The male offspring of FAS dams had elevated serum and liver triglyceride level when fed with HF compared to the male offspring of CON dams. The mRNA and protein expression levels of hepatic *ATGL* and adipose *LPL* were significantly decreased in offspring of FAS dams than in offspring of CON dams. Furthermore, maternal prenatal FAS resulted in elevated DNA methylation levels in the promoter and first exon region of hepatic *ATGL* and adipose *LPL* in offspring. Maternal FAS exacerbated the adverse effects of HF on lipid metabolism in offspring through inducing aberrant DNA methylation levels of hepatic *ATGL* and adipose *LPL*.

## 1. Introduction

Increasing evidence demonstrates that the risk of metabolic diseases in adulthood is determined by not only diet and lifestyle in adulthood, but also the environmental [1], especially nutritional, exposure in early life [2,3,4]. The effects of in utero and early-life nutritional exposure on adult metabolic diseases are thought to be mediated, at least in part, by epigenetic processes [1]. DNA methylation is a stable epigenetic mark [5], which typically acts to repress gene transcription, when located in specific genomic regions, such as promoter or enhancer [6]. Folate, an essential water-soluble vitamin, serves as a key one-carbon source in the methylation cycle [7]. Maternal methyl donor supplementation before and during pregnancy has been found to permanently affect the DNA methylation at some genomic loci in the offspring, resulting in persistent phenotypic modification in offspring [8,9]. However, whether maternal folate supplementation has the potential to alter DNA methylation modification of genes involved in glucose and lipid metabolism in embryo and fetus and then program risk of metabolic diseases in adult offspring is still unclear.

In view of the protective effect of maternal folate supplementation against newborn neural tube defects [10], folate supplementation in the periconceptional period and folate fortification of staple foods has been recommended in many countries [11]. However, higher maternal circulating folate level has also been reported to have negative effects on offspring health. In both the Pune Maternal Nutrition Study and the Parthenon Study, higher maternal circulating folate concentration during pregnancy was associated with higher offspring insulin resistance [12,13]. Rodent studies demonstrated that maternal methyl donor supplementation during pregnancy resulted in elevated colitis susceptibility [14] and risk of allergic airway disease [15] in offspring mice through DNA methylation mechanism. Therefore, maternal folate supplementation during pregnancy seems to be a double-edged sword to offspring health. Both beneficial and potential detrimental effects of maternal prenatal folate supplementation on offspring health should be considered.

In this study, therefore, we aimed to address: (1) whether maternal prenatal folic acid supplementation might result in persistent aberrant DNA methylation modification in glucose and lipid metabolism-related genes and then altered glucose and lipid metabolism in offspring rats; and (2) whether the effects of maternal prenatal folic acid supplementation on offspring glucose and lipid metabolism were further deteriorated by high-fat diet in adulthood, which is a well-known risk factor of adult metabolic diseases. 

## 2. Materials and Methods

### 2.1. Folic Acid Doses Selection and Diets

To better extrapolate our findings to human, the doses of folic acid used in our study were determined based on the folic acid intake in human. Specifically, the recommended folic acid intake for women of childbearing age is 400 μg/day and the tolerable upper intake level for pregnant women is 1000 μg/day [16]. In our study, the AIN-93G diet (2 mg folic acid/kg diet) [17] was used as the control (CON) diet, which is the equivalent of the recommended intake level in human. Accordingly, the folic acid dose in the folic acid supplemental (FAS) diet is 5 mg folic acid/kg diet, 2.5 times the CON diet, which is the equivalent of the tolerable upper intake level in human. The compositions of used diets are shown in Table S1.

### 2.2. Animal Experimentation

All experimental procedures were carried out in accordance with the guidelines for animal use and approved by the Animal Ethics Committee of Harbin Medical University. 

**Figure 1:**
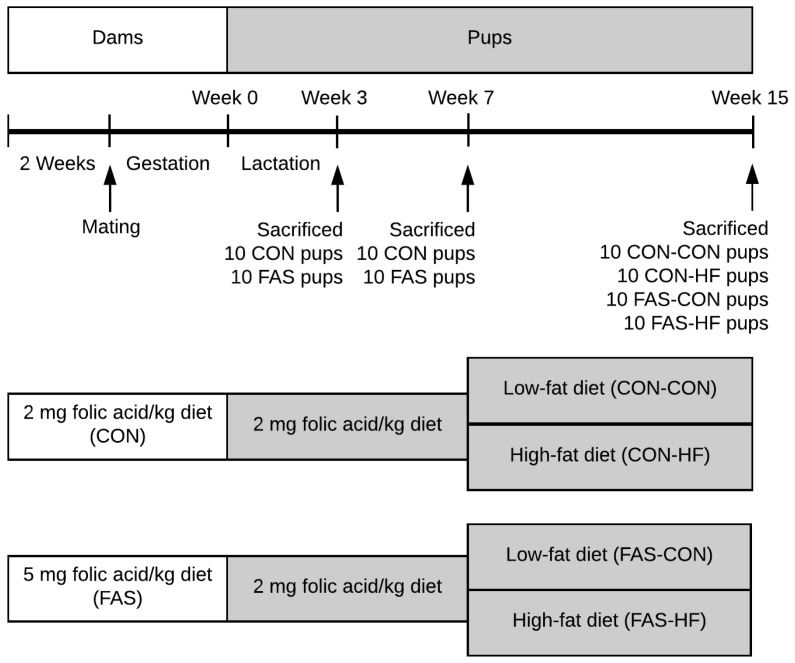
Flow diagram of study design. At weaning (Week 3), 40 CON and 40 FAS male pups were randomly selected from different litters for the following experiments. The rest of male pups and all female pups were sacrificed as this time point. Abbreviations: CON, control; FAS, folic acid supplementation; HF, high-fat diet.

Forty adult female (body weight 180–200 g) and 20 male (body weight 200–220 g) Sprague-Dawley rats were purchased from Vital River Ltd., Beijing, China. All rats were housed individually in wire cages with a standardized environment (21 ± 2 °C, relative humidity 60%, 12-h light/dark cycle and food and water were freely available). After one-week adaptation to the new environment, the female rats were randomly divided into two groups and fed ad libitum CON or FAS diet (Figure 1). After 2 weeks, the females were mated with males (2 females per male) who were fed CON diet throughout the experiment. The female rats were removed and housed individually once the vaginal plugs were observed. The CON dams were fed CON diet during the whole experimental period including 2 weeks before pregnancy, gestation, and lactation. The FAS dams were fed FAS diet in 2 weeks before pregnancy and gestation period, and then CON diet in lactation period. 

After delivery (Week 0), the pups were standardized to six per litter. During the lactation period, all of the dams were fed CON diet. To avoid the potential influence of hormonal fluctuations associated with the female reproductive cycle on outcomes, all the female offspring were removed after weaning (Week 3), and the male offspring were fed CON diet until Week 7. Then, the offspring of both CON and FAS dams were randomly divided into two groups, respectively, of which the rats were fed CON diet (16.4 kJ/g, 12% fat energy, 2 mg folic acid/kg diet) or high-fat (HF) diet (22.0 kJ/g, 60% fat energy, 2 mg folic acid/kg diet) (Table S1) until Week 15. Therefore, the offspring were classified into four groups, i.e., maternal CON-offspring CON (CON-CON), maternal CON-offspring HF (CON-HF), maternal FAS-offspring CON (FAS-CON), and maternal FAS-offspring HF (FAS-HF). 

To examine the metabolic programming changes in the offspring, we collected data at three time points during the offspring development, i.e., at Weeks 3, 7, and 15. At each time point, 10 offspring from different litters were randomly selected from each group and were fasted overnight and anaesthetized with an intraperitoneal injection of sodium pentobarbital (40 mg/kg body weight). The liver, epididymal white adipose tissue, and serum were collected and stored at −80 °C. Totally, 80 male pups were used in this study (Week 3: 10 CON and 10 FAS; Week 7: 10 CON and 10 FAS; and Week 15: 10 CON-CON, 10 CON-HF, 10 FAS-CON, and 10 FAS-HF).

### 2.3. Oral Glucose Tolerance Test (OGTT)

OGTT was performed in offspring at Week 15. Ten rats from each group were fasted for 12 h and then gavaged with 2 g/kg body weight glucose. Blood samples were collected from the tail vein at 0, 30, 60, 90 and 120 min. Blood glucose levels were immediately detected using a Lifescan One Touch Ultra glucometer (Johnson & Johnson, New Brunswick, NJ, USA). The area under curve (AUC) of glucose response was calculated. 

### 2.4. Liver Tissue Triglycerides and Histology and Blood Biochemical Measurements

Hepatic triglyceride (TG) was extracted according to the procedure developed by Folch et al. [18] and detected by a commercial kit (Zhongshengbeikong Biotechnology and Science Inc., Beijing, China). The collected livers were fixed by 10% formalin and embedded by paraffin before sectioning. The liver sections were then stained with hematoxylin and eosin (H&E) to assess hepatic steatosis.

The serum folic acid concentrations were measured by Competitive EIA (enzyme immunoassay) kit (Lifespan Biosciences, Inc., Seattle, WA, USA) with minimum detectable concentrations of 44 pmol/L, intra-assay variation <8%, and inter-assay variation <10%. The serum homocysteine concentrations were measured by Sandwich ELISA (enzyme-linked immunosorbent assay) kit (Lifespan Biosciences, Inc., Seattle, WA, USA) with minimum detectable concentrations of 0.26 μmol/L, intra-assay variation <10%, and inter-assay variation <12%. The fasting serum levels of glucose, total cholesterol (TC), TG, high density lipoprotein cholesterols (HDL), low density lipoprotein cholesterols (LDL), alanine aminotransferase (ALT), and aspartate aminotransferase (AST) were measured using standard enzymatic methods in an auto-analyzer (AUTOLAB PM 4000, AMS Corporation, Rome, Italy).

### 2.5. Quantitative Real-Time PCR and Western Blotting

**Figure 2:**
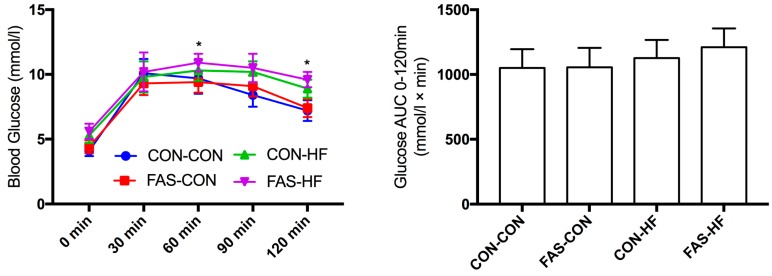
Blood glucose concentrations and the area under curve of blood glucose during oral glucose tolerance test. Values are means with standard deviations (*n* = 10). * The difference between FAS-HF and FAS-CON/CON-CON is significant, *p* < 0.05.

In this study, we found that maternal prenatal FAS resulted in elevated serum TG concentration and lipid deposition in liver in offspring fed with HF diet (Table 1 and Figure 2). To elucidate which lipid metabolic pathway in offspring was affected by maternal FAS, we examined the mRNA expression of 23 representative genes, which are involved in the determinant processes of lipid metabolism, such as fatty acid biosynthesis, fatty acid degradation, adipocytokine signaling pathway, lipolysis regulation, glycerolipid metabolism, and fatty acid transport. The mRNA expressions of all 23 genes were detected using quantitative real-time PCR at Week 3 (weaning). The genes of which mRNA expression was significantly affected by maternal FAS were selected for the following experiments including Western blotting and DNA methylation determination. 

Total mRNA was extracted from liver samples with Trizol (Invitrogen, Life Technologies Corp., Carlsbad, CA, USA). cDNA was synthesized using a High Capacity cDNA Reverse Transcription Kit (Applied Biosystems Inc., Foster City, CA, USA). The mRNA levels of 23 lipid metabolism-related genes were determined using the Applied Biosystems 7500 Fast Real-Time PCR System with SYBR Green PCR Master mix (Applied Biosystems Inc., Foster City, CA, USA). The thermal cycling program was 50 °C for 2 min, followed by 95 °C for 10 min for 1 cycle, then 95 °C for 15 s, followed by 60 °C for 1 min for 40 cycles. To verify the accuracy and specificity, all reactions were performed in triplicate, followed by melting curve analysis. The genes’ names and corresponding primers are shown in Table S2. β-actin was used as a reference gene for normalization in each reaction. Relative expression differences were calculated using the 2(-Delta Delta C(T)) method [19].

The protocol of Western blotting in our laboratory has been described previously [20]. In this study, the protein expression levels of Adipose triglyceride lipase (*ATGL*) in hepatic tissue and Lipoprotein lipase (*LPL*) in white adipose tissue were detected. The antibodies were as follows: *ATGL* and *LPL* antibodies (Cell Signaling Technology, Beverly, MA, USA), β-actin and secondary antibody (Santa Cruz Biotechnology, Dallas, TX, USA). 

### 2.6. DNA Methylation Determination

The methylation status of CpG sites within the *ATGL* and *LPL* promoters and first exon regions (Figure S1) were examined by bisulfite sequencing PCR (BSP). Genomic DNA was extracted from liver and adipose tissue and bisulfite-converted using EpiTect Fast DNA Bisulfite Kit (Qiagen). Bisulfite-treated DNA was amplified using *ATGL* and *LPL* primers (Table S3). The PCR products were ligated to the pGEM-T vector (Promega, Madison, WI, USA) and transformed into competent cells. The cells that carry the ligated vectors were selected on agar plates containing ampicillin. Ten positive colonies were randomly selected for each rat and grown in LB medium. Plasmids were extracted by using the QIAprep Spin Miniprep Kit (Qiagen) and subjected to standard sequencing analysis. DNA methylation status was determined by the sequencing results. Basically, all unmethylated cytosines (C) have been convert to thymine (T) after bisulfite treatment and the presence of a C-peak indicates the presence of 5-methylcytosine (5mC) in the genome. DNA methylation level (%) at each CpG locus for one rat was calculated as: (the number of “C”s at one locus among the 10 colonies/10) × 100. 

### 2.7. Statistical Analysis

Data are presented as means ± standard deviations (SD). The normal distribution of the variables was tested by the Kolmogorov-Smirnov and the Shapiro-Wilk tests. The data with skewed distribution were log-transformed to fit normal distribution before conducting the following statistical analyses. The data generated at Week 3 and Week 7 were analyzed by two independent samples *t*-test. The data generated at Week 15 were analyzed by two-way analysis of variance (ANOVA) (maternal FAS × offspring HF), followed by Tukey’s post hoc test. Statistical analyses were performed with SPSS 20.0 (IBM Co., Armonk, NY, USA), with α = 0.05. 

## 3. Results

### 3.1. Maternal and Offspring Food Intake and Serum Folic Acid Levels

As shown in Figure S2, maternal and offspring daily food intake was not significantly different between different groups. Compared with the CON dams, the FAS dams had significantly higher serum folic acid and lower homocysteine (Hcy) levels at delivery (Figure S3). 

### 3.2. Offspring Body Weight, Glucose and Lipid Metabolism at 15 Weeks

We did not find maternal prenatal FAS had significant effects on offspring body weight (Table 1 and Figure S4), fasting glucose (Table 1), and the AUCs of blood glucose during OGTT (Figure 2) at 15 weeks.

**Figure 3:**
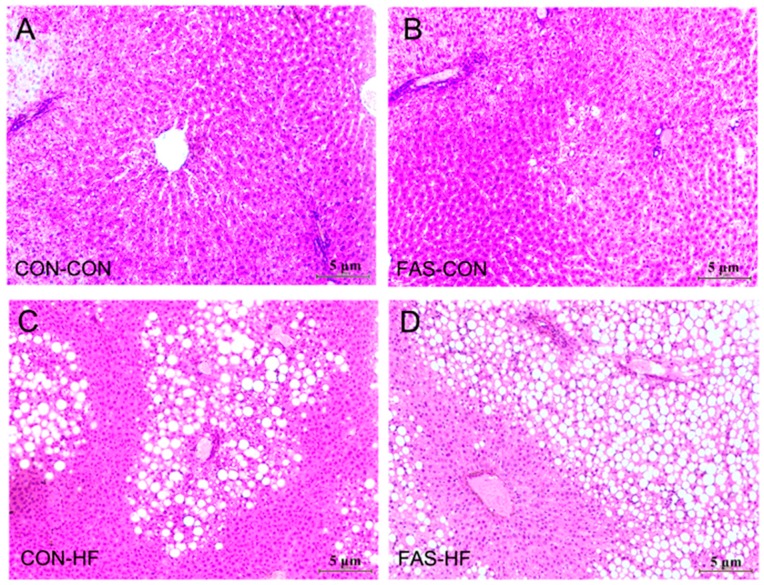
H&E stains of representative liver sections of male: CON-CON (**A**); FAS-CON (**B**); CON-HF (**C**); and FAS-HF (**D**) offspring at 15 weeks (40×).

Maternal prenatal FAS resulted in elevated serum and liver TG (*p* < 0.001) and liver body weight ratio (*p* = 0.02) in 15-week-old offspring (Table 1). Moreover, maternal FAS and offspring HF had significant interactive effects on offspring serum and liver TG and liver body weight ratio (Table 1), which means the effects of maternal FAS on these outcomes were exacerbated by offspring HF. The liver pathological section results further confirmed the interactive effects of maternal FAS and offspring HF on liver lipid deposition (Figure 3). However, such interactive effects were not found on serum HDL, LDL, ALT, and AST, although offspring HF had significant effects on these lipid metabolism markers.

### 3.3. Expression of Lipid Metabolic Related Genes in Liver and White Adipose Tissue

**Figure 4:**
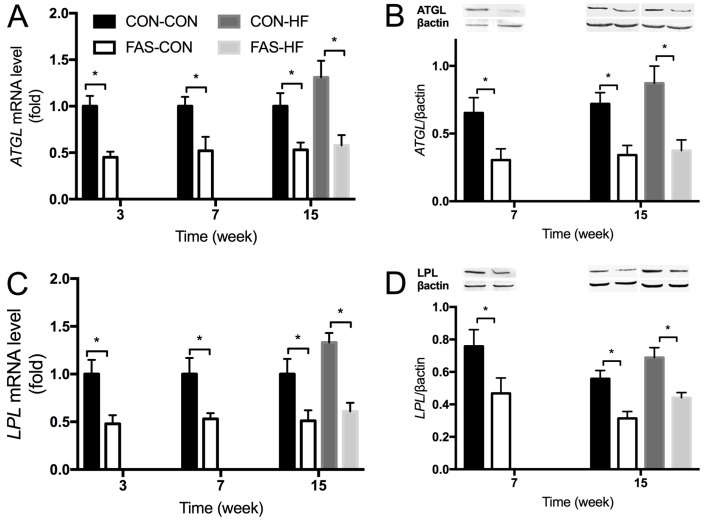
The hepatic: *ATGL* mRNA (**A**); and protein (**B**) levels; and white adipose *LPL* mRNA (**C**); and protein (**D**) levels in the male offspring at different time points. Representative Western blot photographs are shown (**B**,**D**). Values are means with standard deviations (*n* = 10). * The difference between the two groups indicated by the line segment is significant, *p* < 0.05.

To elucidate the underlying mechanisms of maternal FAS affecting offspring lipid metabolism, mRNA expression of 23 genes involved in lipid metabolism in liver and white adipose tissue were firstly detected in three-week-old offspring. The results showed that the expression of hepatic *ATGL* and adipose *LPL* in offspring born from FAS dams was significantly lower than in offspring of CON dams (Table 2). Furthermore, these differences persistently existed at Week 7 and were not changed by the HF intervention at Week 15 (Figure 4A,C). The protein expression of *ATGL* in liver and *LPL* in white adipose tissue was also consistent with the mRNA expression results (Figure 4B,D).

### 3.4. DNA Methylation of ATGL and LPL

**Figure 5:**
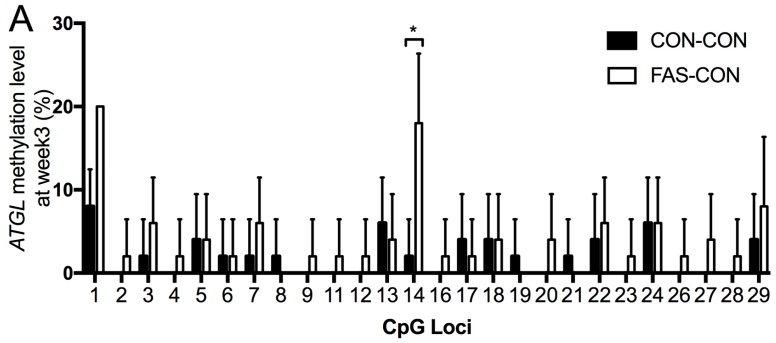

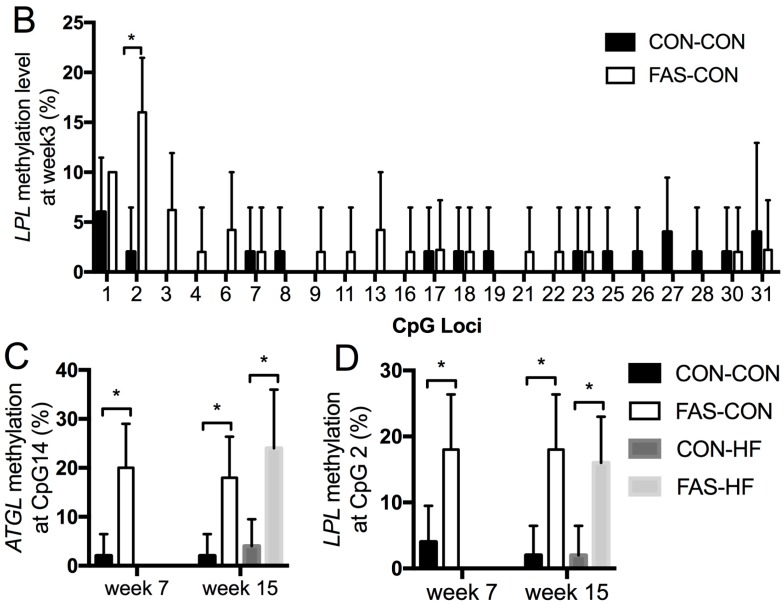
DNA methylation of: hepatic *ATGL* (**A**,**C**); and adipose *LPL* (**B**,**D**) at Weeks of 3, 7, and 15. Values are means with standard deviations (*n* = 10). * The difference between the two groups indicated by the line segment is significant, *p* < 0.05.

To determine whether the mRNA and protein expression differences were caused by DNA methylation alteration, we measured the methylation levels of the CpG sites in the CpG islands located within the promoter and/or first exon regions of *ATGL* and *LPL* genes (Figure S3). At Week 3, among the 29 CpG loci of *ATGL* gene in liver, the DNA methylation level at CpG 14 in pups from FAS dams were significantly higher than that in pups from the CON dams (Figure 5A). Similarly, the FAS pups also had higher DNA methylation level at CpG 2 of *LPL* gene in white adipose tissue than the CON pups at three weeks old (Figure 5B). These DNA methylation differences also exist at Week 7 and 15, regardless of the HF intervention at Week 15 (Figure 5C,D). 

## 4. Discussion

Our findings demonstrate that maternal prenatal FAS leads to elevated serum and liver TG concentration in adult offspring and this effect can be further exaggerated by the offspring high-fat diet. Moreover, maternal prenatal FAS induced persistently decreased expression of hepatic *ATGL* and adipose *LPL*, which was potentially caused by elevated DNA methylation levels within *ATGL* and *LPL* promoter/first exon region. Taken together, maternal prenatal FAS may program lipid metabolism in adult offspring through regulating hepatic *ATGL* and adipose *LPL* methylation.

To prevent the newborns from neural tube defects, pregnant women are mandatorily asked to supplement folic acid during periconceptional period in many countries. However, in addition to reducing the risk of neural tube defects, the effects of maternal FAS on offspring glucose and lipid metabolism remain unclear. Some studies reported a protective effect of maternal FAS on offspring metabolism, whereas some others showed adverse effect or no effect. The discordance among the published results may be caused by the used folic acid doses, the period of intervention, the gender of offspring, etc. 

Firstly, the used folic acid dose is the main concern when interpreting study results. Generally, low-dose folic acid supplementation exerted beneficial effects on metabolism, whereas high doses had adverse or no effect. Chmurzynska et al. reported that maternal FAS diet (5 vs. 2 mg folic acid/kg diet) resulted in reduced circulating glucose and total cholesterol concentration in offspring [21]. Cho et al. found that high folate gestational diet (20 vs. 2 mg folic acid/kg diet) resulted in increased body weight and food intake and impaired glucose response to insulin in the offspring [22]. A previous study from our lab reported that maternal high FAS (40 vs. 2 mg folic acid/kg diet) led to increased insulin resistance in male mouse offspring fed a high-fat diet at weaning [23]. Such inconsistency also exists in human studies. One study reported that low-dose folic acid supplementation had beneficial effects on blood lipids in adults [24]. In contrast, the high maternal dietary folate intake at 32 weeks of pregnancy was not found to be associated with offspring body weight at nine years of age in a UK cohort study [25]. Two Indian cohorts revealed that higher maternal plasma or red blood cell folate concentrations during pregnancy were positively associated with adiposity and insulin resistance in offspring at ages of 6, 9.5, and 13.5 years [12,13]. Secondly, the period of folic acid intervention should also be considered when evaluating the effects of FAS. For example, maternal FAS (5 mg folic acid/kg diet) during pregnancy was reported to have beneficial effect on glucose and lipid metabolism [21]. However, Burdge et al. found that FAS diet (5 vs. 1 mg folic acid/kg diet) during the juvenile-pubertal period increased body weight, and hepatic and plasma TG concentration irrespective of maternal diet [26]. Third, sex difference is another issue. Hoile et al. found that maternal FAS diet (5 vs. 1 mg folic acid/kg diet) during pregnancy increased plasma glucose concentration in females, but not in males [27]. 

Taken together, both existing animal and human studies demonstrate that the long-term effect of maternal prenatal FAS on offspring metabolism can be modified by many factors, the entire study design should be considered before drawing conclusion. With our current study design, the results showed that maternal FAS (5 mg folic acid/kg diet) during pregnancy and two weeks before pregnancy resulted in increased serum and liver TG levels in male offspring, especially in offspring fed a HF diet from weaning.

To clarify the underlying mechanism of maternal prenatal FAS affecting offspring lipid metabolism, we dynamically detected the mRNA expression of 23 well-known genes involved in lipid metabolism in liver and white adipose tissue at different stages of offspring development, i.e., at Weeks 3, 7, and 15. Among the 23 lipid metabolism-related genes, the mRNA and protein expression of *ATGL* in liver and *LPL* in white adipose tissue were found to be persistently decreased in the male offspring of dams fed with FAS diet. *ATGL* has been identified as a major lipase that regulates triacylglycerol turnover and fatty acid signaling and partitioning in adipose tissue [28] and liver [29]. Knockdown of hepatic *ATGL* was reported to cause steatosis in mice and decreased hydrolysis of triacylglycerol in primary hepatocyte [29]. *LPL* is reported as an enzyme which catalyze the rate-limiting step of triglycerides in peripheral tissues, such as adipose tissue [30]. One recent human study showed that a newborn with monogenic *LPL* deficiency had severe hypertriglyceridemia [31]. Therefore, the down-regulated *ATGL* in liver and *LPL* in white adipose tissue may mediate the effect of maternal prenatal FAS on offspring lipid metabolism in our study.

Folic acid, or folate, provides the methyl group in the synthesis pathway for *S*-adenosylmethionine, which serves as a primary methyl group donor for the majority of methylation reactions, including DNA methylation, which is essential for the epigenetic control of gene transcription [32]. Recent animal and human studies have demonstrated that maternal FAS and plasma folate concentration during pregnancy can influence DNA methylation and gene expression in offspring [33,34]. Our results firstly showed that the DNA methylation levels of *ATGL* and *LPL* were increased in offspring of dams fed with FAS diet, which supports the notion that maternal prenatal FAS may result in DNA methylation changes in hepatic *ATGL* and adipose *LPL* and then disturbed lipids metabolism in offspring.

Although it is the first time to report that maternal folic acid supplementation resulted in DNA methylation changes in hepatic *ATGL* and adipose *LPL*, previous studies demonstrated the possibility that maternal folic acid levels could alter DNA methylation levels in the offspring [35]. Early development, when the DNA methylation patterns of fetus are erased and reset and could impact later health outcomes in the offspring [36], is particularly susceptible to maternal folate intake. One recent study profiled genome-wide changes in DNA methylation and gene expression in mouse liver caused by maternal folate depletion and high fat intake post-weaning. The affected genes were involved in many pathways including glycolysis and gluconeogenesis, amino acid metabolism, and fatty acid biosynthesis, which is in support of our findings in this study. Additionally, the combined dietary insult (i.e., maternal folate depletion followed by high fat post-weaning) reportedly caused the largest expression change for most genes [37]. In our study, maternal FAS combined with offspring HF also had the most effects on lipid metabolism although did not show additive effects on DNA methylation changes. In addition, in a recent epigenome-wide meta-analysis of 1988 newborns, Bonnie et al. reported the associations between maternal plasma folate during pregnancy and DNA methylation in cord blood at 443 CpGs, which get involved in multiple biological pathways [34]. It seems that the effects of maternal folate on offspring epigenome are extensive. The beneficial or detrimental effects of maternal FAS on offspring health, to some extends, depend on what genes are regulated by DNA methylation changes induced by maternal FAS, although it is still unclear how the DNA methylation at specific loci in offspring is affected by maternal FAS.

In conclusion, compared to CON (2 mg folic acid/kg diet), maternal prenatal FAS (5 mg folic acid/kg diet) could result in persistent DNA methylation changes in hepatic *ATGL* and adipose *LPL* in offspring, which exacerbated the effect of HF diet on lipid metabolism in adult offspring. 

## Figures and Tables

**Table 1:** Metabolic variables of male offspring at 15 weeks of age in different groups (*n* = 10).

	CON-CON	FAS-CON	CON-HF	FAS-HF	*p*_1_	*p*_2_	*p*_3_
Body Weight, g	460.3 ± 23.8	468.2 ± 23.6	565.4 ± 24.3	583.7 ± 24.9	0.48	<0.001	0.5
Fasting glucose, mmol/L	4.62 ± 0.81	4.52 ± 0.54	4.93 ± 0.79	5.62 ± 0.76	0.17	0.002	0.07
Total cholesterol, mmol/L	1.33 ± 0.66	1.41 ± 0.48	1.92 ± 0.74	2.20 ± 0.38	0.29	<0.001	0.55
Triglyceride, mmol/L	0.71 ± 0.09	0.84 ± 0.12	1.24 ± 0.37	1.92 ± 0.27	<0.001	<0.001	0.001
High density lipoprotein cholesterol, mmol/L	0.82 ± 0.07	0.82 ± 0.06	0.92 ± 0.13	0.96 ± 0.08	0.59	<0.001	0.45
Low density lipoprotein cholesterol, mmol/L	0.31 ± 0.05	0.32 ± 0.06	0.47 ± 0.10	0.53 ± 0.05	0.11	<0.001	0.26
Alanine aminotransferase, IU/L	34.74 ± 4.23	34.61 ± 3.55	70.49 ± 7.83	77.84 ± 7.87	0.06	<0.001	0.06
Aspartate aminotransferase, IU/L	128.02 ± 15.26	127.34 ± 13.34	148.03 ± 19.29	155.67 ± 15.43	0.46	<0.001	0.38
Liver body weight ratio, %	2.36 ± 0.15	2.39 ± 0.15	3.17 ± 0.25	3.47 ± 0.27	0.02	<0.001	0.04
Liver triglyceride, mmol/g	39.07 ± 6.11	52.60 ± 7.36	85.51 ± 15.81	137.98 ± 16.63	<0.001	<0.001	<0.001

Values are means with standard deviations. *p*_1_ indicates the effect of maternal folic acid supplement; *p*_2_ indicates the effect of high-fat diet in adulthood in offspring; *p*_3_ indicates the interaction between maternal folic acid supplement and offspring high-fat diet.

**Table 2:** The relative mRNA expression of genes involved in hepatic and adipose lipid metabolism in three-week-old male offspring of dams supplemented with folic acid compared with their control counterparts (*n* = 10).

Genes	Fold Changes in Liver	Fold Changes in White Adipose Tissues
*Acetyl-CoA carboxylase*	1.06 ± 0.09	1.35 ± 0.15
*Acyl-CoA oxidase 1*	1.29 ± 0.15	0.93 ± 0.09
*Adiponectin*	1.32 ± 0.12	1.07 ± 0.09
*Adiponectin receptor*	0.82 ± 0.07	1.15 ± 0.1
*AMP-activated protein kinase 1*	0.93 ± 0.09	0.86 ± 0.09
*AMP-activated protein kinase 2*	1.13 ± 0.12	1.45 ± 0.12
*Adipose triglyceride lipase*	0.45 ± 0.06 ^*^	0.91 ± 0.10
*Cluster of Differentiation 36*	1.09 ± 0.1	1.27 ± 0.15
*Carnitine palmitoyltransferase 1*	1.04 ± 0.12	1.12 ± 0.12
*Diglyceride acyltransferase 1*	0.83 ± 0.1	1.09 ± 0.1
*Diglyceride acyltransferase 2*	1.46 ± 0.16	1.16 ± 0.14
*Fatty acid-binding protein*	0.81 ± 0.09	1.28 ± 0.15
*Fatty acid synthase*	1.14 ± 0.12	1.31 ± 0.15
*Hormone-sensitive lipase*	1.32 ± 0.11	1.35 ± 0.13
*Leptin*	0.88 ± 0.1	1.16 ± 0.11
*Lipoprotein lipase*	0.82 ± 0.09	0.53 ± 0.06 ^*^
*Perilipin*	1.05 ± 0.09	0.93 ± 0.09
*Peroxisome proliferator-activated receptor α*	1.11 ± 0.11	0.88 ± 0.08
*Peroxisome proliferator-activated receptor γ*	1.21 ± 0.11	1.31 ± 0.18
*Resistin*	0.84 ± 0.09	0.82 ± 0.07
*Stearoyl-CoA desaturase-1*	1.11 ± 0.11	0.88 ± 0.08
*NAD-dependent deacetylase sirtuin-3*	0.82 ± 0.09	0.78 ± 0.09
*Sterol regulatory element-binding protein 1c*	1.18 ± 0.18	0.85 ± 0.08

Values are means with standard deviations. The gene expression fold changes were calculated as: the normalized relative mRNA expression in FAS/the normalized relative mRNA expression in CON. * The fold change is statistically significant, *p* < 0.05.

## Supplementary Materials

The following are available online at www.mdpi.com/2072-6643/9/9/935/s1, Table S1. Compositions of the diets; Table S2. mRNA primers of genes related to lipid metabolism; Table S3. Methylation primers of *ATGL* and *LPL*; Figure S1. Schematic diagram of the CpG loci in *ATGL* and *LPL*; Figure S2. Food intake of dams and pups in different groups. Values are means with standard deviations; Figure S3. Serum folic acid and homocysteine levels of dams at delivery. Values are means with standard deviations. * The difference between the two groups indicated by the line segment is significant, *p* < 0.05; Figure S4. Body weight changes of male offspring in different groups. Values are means with standard deviations. * The difference between CON-HF/FAS-HF and CON-CON/FAS-CON is significant, *p* < 0.05.
