# Supplementary material for: Maternal Prenatal Folic Acid Supplementation Programs Offspring Lipid Metabolism by Aberrant DNA Methylation in Hepatic ATGL and Adipose LPL in Rats
Source: Nutrients. 2017 Aug 26;9(9):935. doi: 10.3390/nu9090935 (PMC5622695; doi:10.3390/nu9090935)
Supplement: Supplementary file 1 [file nutrients-09-00935-s001.pdf]

nutrients

MDPI

Supplementary Materials

Table S1. Compositions of the diets.

Diet Component	CON Diet	FAS Diet	HF Diet
Maize starch (g/kg)	330	330	130
Dextrin (g/kg)	165	165	65
Sucrose (g/kg)	165	165	65
Soya bean (g/kg)	48	48	64
Lard (g/kg)	4	4	283
Casein (vitamin free) (g/kg)	200	200	270
L-Cystine (g/kg)	3	3	4
Inulin (g/kg)	20	20	27
Powdered cellulose (g/kg)	20	20	27
Mineral mix (g/kg) †	35	35	48
Vitamin mix (g/kg) ‡	10	10	14
Folic acid (mg/kg)	2	5	2
Choline bitartrate (g/kg)	2	2	2·7
Energy density (kJ/g diet)	16·4	16·4	22·0
Fat (energy%)	11.9	11.9	59.4
Carbohydrate (energy%)	67.3	67.3	19.8
Protein (energy%)	20.8	20.8	20.8

CON, control; FAS, folic acid supplementation; HF, high fat; † AIN-93G mineral mix; ‡ AIN-93G vitamin mix except for folic acid.

Table S2. mRNA primers of genes related to lipid metabolism.

Gene	Primer Sequence
Acetyl-CoA carboxylase	F:5'-TTTTCGATGTCCTCCCAAACTTTT-3'
	R:5'-GCTCATAGGCGATATAAGCTCTTC-3'
Acyl-CoA oxidase 1	F:5'-CACGCAATAGTTCTGGCTCA-3'
	R:5'-ACCTGGGCGTATTTCATCAG-3'
Adiponectin	F:5'-GGGACAACAATGGACTCTATGC-3'
	R:5'-GTTCTTTGATTCTCGGGGCTA-3'
Adiponectin receptor 1	F:5'-GGCTGAAAGACAATGACTAC-3'
	R:5'-TCAAGATTCCCAGAAAGAG-3'
AMP-activated protein kinase 1	F:5'-CCGTCTGATATTTTCATGGTCA-3'
	R:5'-ACTCTCCTTTTCGTCCAACCT-3'
AMP-activated protein kinase 2	F:5'-CCGGAGGTCATCTCAGGA-3'
	R:5'-AGGGCATACAGGATAACACCA-3'
Adipose triglyceride lipase	F:5'-CACTTTAGCTCCAAGGATGA-3'
	R:5'-TGGTTCAGTAGGCCATTCCT-3'
Cluster of Differentiation 36	F:5'-CGGCGATGAGAAAGCAGAA-3'
	R:5'-CCAGGCCCAGGAGCT TTATT-3'
Carnitine palmitoyltransferase 1	F:5'-GCCCATGTTGTACAGCTTCCA-3'
	R:5'-AGTCTTCTTCCTTCATCAGTGGC-3'

Diglyceride acyltransferase 1	F:5'-TTTCTGCTACGGCGGGTTCTTGAG-3'
	R:5'-ACCGGTTGCCCAATGATGAGTGTC-3'
Diglyceride acyltransferase 2	F:5'-GGAGGCCACCGAAGTTAGCAAGAA-3'
	R:5'-AGCCCCCAGGTGTCAGAGGAGAAG-3'
Fatty acid-binding protein	F: 5'-GACCTCATCCAGAAAGGGAAG-3'
	R: 5'-CACGGACTTTATGCCTTTGAA-3'
Fatty acid synthase	F:5'-TCTCTGGTGGTGTCTACATTTCG-3'
	R:5'-GCAGGATAGCACTCTCAGACAG-3'
Hormone-sensitive lipase	F:5'-TCTCTGGTGGTGTCTACATTTCG-3'
	R:5'-GCAGGATAGCACTCTCAGACAG-3'
Leptin	F:5'-ATTTCACACACGCAGTCGGTATCCG-3'
	R:5'-CCAGCAGATGGAGGAGGTC-3'
Lipoprotein lipase	F:5'-TATGGCACAGTGGCTGAAAG-3'
	R:5'-CTGACCAGCGGAAGTAGGAG-3'
Perlipin	F:5'-GTACACTATGTCCCGCTTCC-3'
	R:5'-CCACCTCTGCTGGAGGATTA-3'
Peroxisome proliferator-activated receptor α	F:5'-TCATACTCGCAGGAAAGACT-3'
	R:5'-ACCTCTGCCTCCTTGTTTTC-3'
Peroxisome proliferator-activated receptor γ	F:5'-CCCATTCCTTTGACATCAAACC-3'
	R:5'-ATTGTGAGACATCCCCACAGC-3'
Resistin	F:5'-TTCCTTGTCCTGGGGCTGCTGG-3'
	R:5'-CATCCCTCGGCAGCACTCGG-3'
Stearoyl-CoA desaturase-1	F:5'-TCACCTTGAGAGAAGAATTAGCA-3′
	R:5'-TTCCCATTCCCTTCACTCTGA-3′
NAD-dependent deacetylase sirtuin-3	F:5'-TGCACGGTCTGTCGAAGGTC-3′
	R:5'-ATGTCAGGTTTCACAACGCCAGTA-3′
Sterol regulatory element-binding protein 1c	F:5'-GCAACACTGGCAGAGATCTACGT-3'
	R:5'-TGGCGGGCACTACTTAGGAA-3'
β-Actin	F: 5′-AGGGAAATCGTGCGTGAC-3′
	R: 5′-CGCTCATTGCCGATAGTG-3′

Table S3. Methylation primers of *ATGL* and *LPL.*

Gene	Sequence
Adipose triglyceride lipase	F: 5'-ATGGGGGTATTTATTATTGT-3′
(ATGL)	R: 5'-ACCACTCCAATATAATAAAC-3′
Lipoprotein lipase	F: 5'-TATGTGTTTTAAGGTTTTTTTGG-3′
(LPL)	R: 5'-GGAGTTTGGTTTTAGAGTTTGA-3′

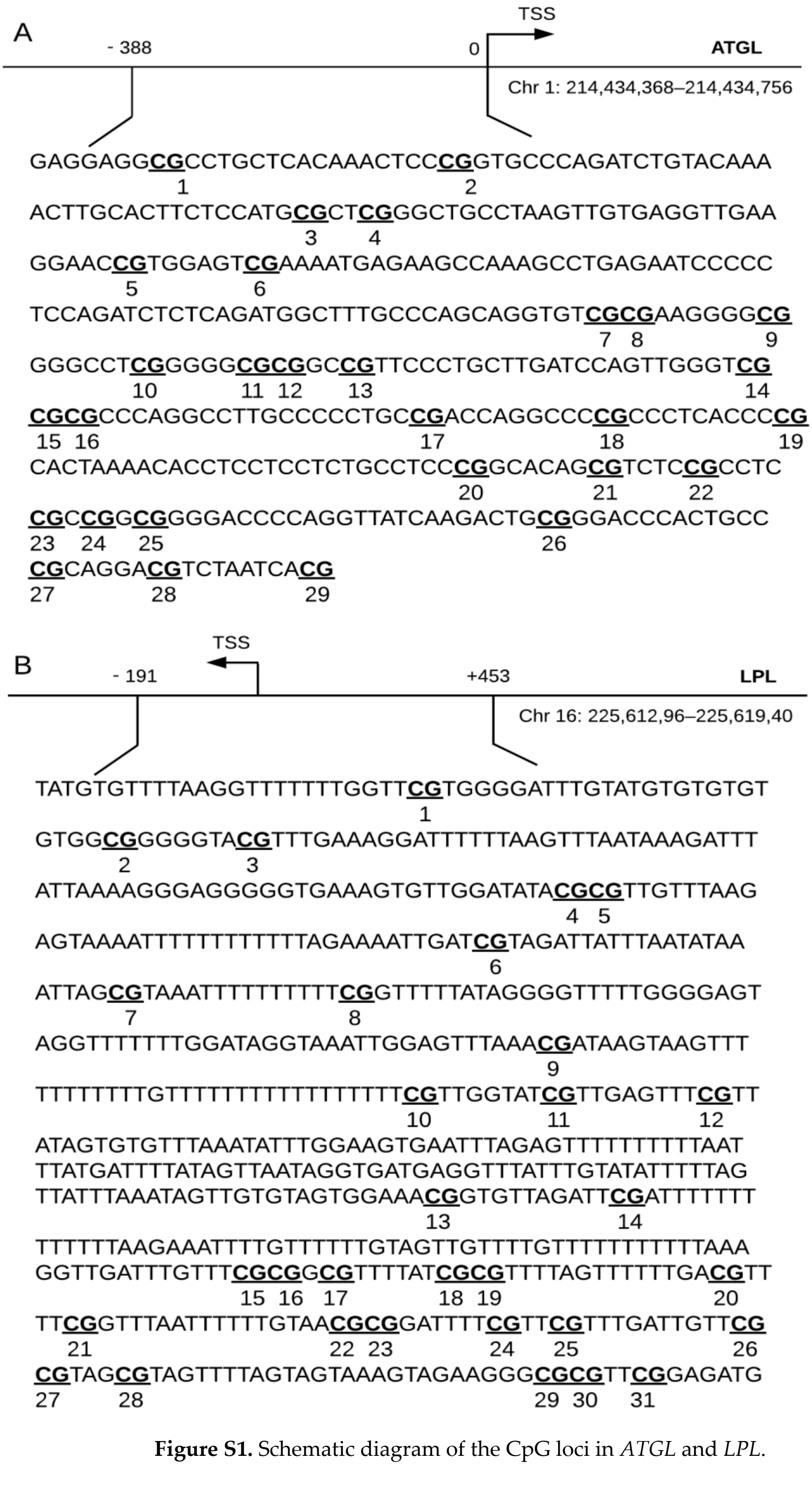

Figure S1. Schematic diagram of the CpG loci in *ATGL* and *LPL*.

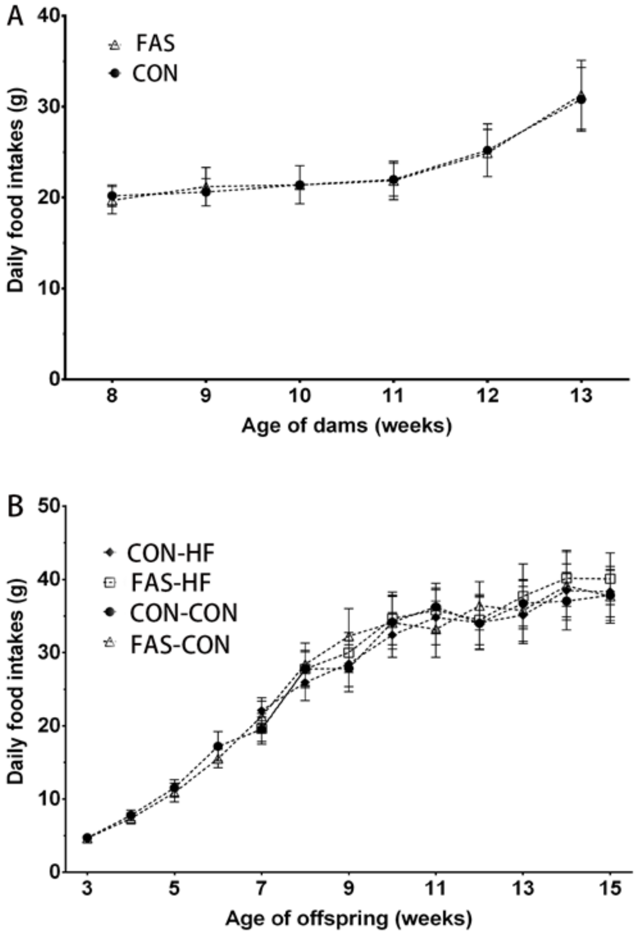

Figure S2. Food intake of dams and pups in different groups. Values are means with standard deviations.

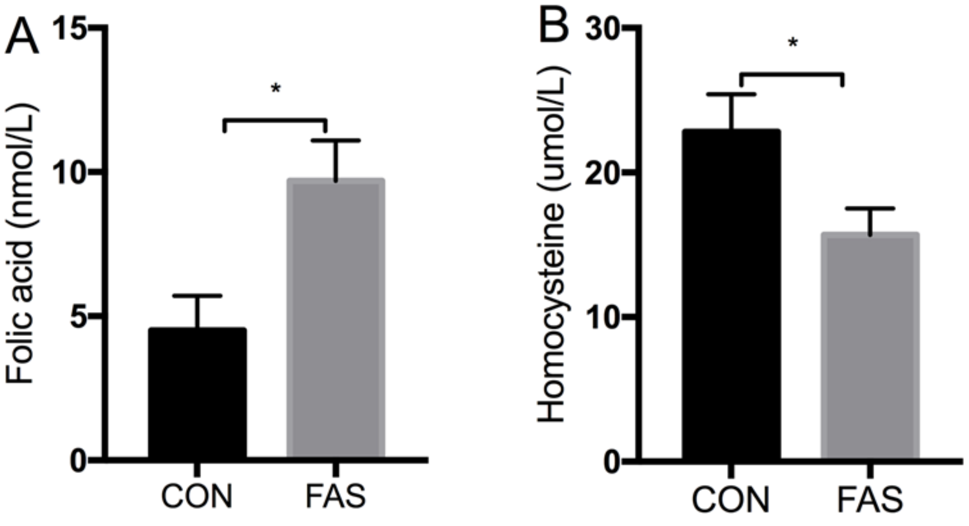

Figure S3. Serum folic acid and homocysteine levels of dams at delivery. Values are means with standard deviations. * The difference between the two groups indicated by the line segment is significant, $p < 0.05$.

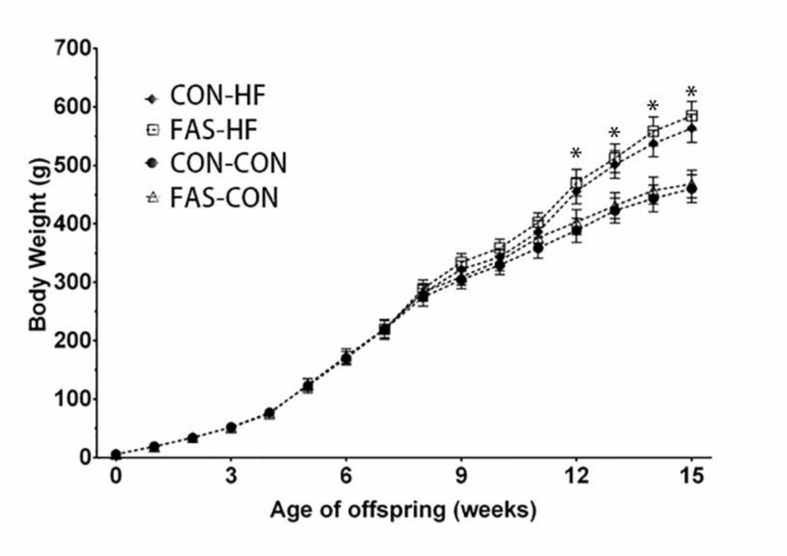

Figure S4. Body weight changes of male offspring in different groups. Values are means with standard deviations. * The difference between CON-HF/FAS-HF and CON-CON/FAS-CON is significant, $p < 0.05$.
